# Association of Increased Receptor-Binding Avidity of Influenza A(H9N2) Viruses with Escape from Antibody-Based Immunity and Enhanced Zoonotic Potential

**DOI:** 10.3201/eid2501.180616

**Published:** 2019-01

**Authors:** Joshua E. Sealy, Tahir Yaqub, Thomas P. Peacock, Pengxiang Chang, Burcu Ermetal, Anabel Clements, Jean-Remy Sadeyen, Arslan Mehboob, Holly Shelton, Juliet E. Bryant, Rod S. Daniels, John W. McCauley, Munir Iqbal

**Affiliations:** The Pirbright Institute, Pirbright, UK (J.E. Sealy, T.P. Peacock, P. Chang, A. Clements, J.-R. Sadeyen, H. Shelton, M. Iqbal);; University of Veterinary and Animal Sciences, Lahore, Pakistan (T. Yaqub, A. Mehboob);; The Francis Crick Institute, London (B. Ermetal, R.S. Daniels, J.W. McCauley);; Fondation Mérieux, Lyon, France (J.E. Bryant)

**Keywords:** influenza A(H9N2) virus, viruses, influenza, receptor-binding avidity, virus escape, antibody-based immunity, respiratory infections, enhanced zoonotic potential, zoonoses, Pakistan

## Abstract

We characterized 55 influenza A(H9N2) viruses isolated in Pakistan during 2014–2016 and found that the hemagglutinin gene is of the G1 lineage and that internal genes have differentiated into a variety of novel genotypes. Some isolates had up to 4-fold reduction in hemagglutination inhibition titers compared with older viruses. Viruses with hemagglutinin A180T/V substitutions conveyed this antigenic diversity and also caused up to 3,500-fold greater binding to avian-like and >20-fold greater binding to human-like sialic acid receptor analogs. This enhanced binding avidity led to reduced virus replication in primary and continuous cell culture. We confirmed that altered receptor-binding avidity of H9N2 viruses, including enhanced binding to human-like receptors, results in antigenic variation in avian influenza viruses. Consequently, current vaccine formulations might not induce adequate protective immunity in poultry, and emergence of isolates with marked avidity for human-like receptors increases the zoonotic risk.

Since their first detection in China in 1992, avian influenza A(H9N2) viruses of the G1 and BJ94 lineages have become enzootic to poultry in Asia and parts of Africa (*1*–*3*). These viruses frequently cause outbreaks in these regions and sporadic outbreaks in Europe and North America (*4*–*9*). H9N2 viruses cause moderate illness and death rates in domestic poultry, leading to major economic burden to small-scale and large-scale poultry industries, and increased risk for zoonotic infection (*6*,*10*,*11*).

Severe illness in humans infected with this virus is rare, but seroepidemiologic data suggest that infection might be most common in those working at the human–animal interface (*12*–*14*). It is evident that there is a major genetic host barrier between currently circulating H9N2 viruses and humans, despite detection of molecular markers of mammalian tropism in avian isolates (*15*,*16*). Adaptation to humans requires permissive mutations throughout the genome of avian influenza viruses that affect multiple factors, such as receptor binding, pH stability, virus polymerase activity, innate immune responses, and viral egress (*17*–*21*).

Hemagglutinin (HA) and neuraminidase (NA) play critical roles in overcoming this genetic host barrier, as shown by recent zoonotic infections with H7N9, H10N8, and H5Nx viruses, all containing H9N2 internal genes, compared with the remarkable dearth of reported human infections with H9N2 viruses, despite their higher incidence in poultry (*15*,*22*,*23*). Efficient virus replication is dependent, in part, on the concerted activities of HA and NA binding and eluting cells and is a balance maintained by matching HA substitutions that alter receptor-binding avidity with changes to NA stalk length and sialidase activity (*24*–*26*).

HA is a membrane-bound homotrimer that becomes functionally active through proteolytic cleavage of precursor HA0 into HA1 and HA2 polypeptides. The HA1 globular head domain facilitates host-cell binding, which initiates infection, and the HA2 fusion peptide, primed during cleavage activation, facilitates pH-dependent fusion between virus and host-cell membranes (*27*). Human-to-human transmissible influenza viruses typically display preferential binding to glycans containing terminal α2,6-linked sialic acids (SAs), which are prevalent in the upper respiratory tract of humans (*19*,*28*,*29*); avian-origin viruses typically bind preferentially to glycans having terminal α2,3-linked SAs, which are found throughout the avian gastrointestinal and respiratory tracts (*30*). The 3′-sialyllactosamine and 6′-sialyllactosamine receptor analogs are commonly used in influenza receptor-binding assays representing α2,3-linked (3SLN) and α2,6-linked (6SLN) SAs (*31*). The switch in binding preference of H9N2 viruses from avian-like α2,3-linked to human-like α2,6-linked SAs has been attributed to amino acid substitutions in the receptor-binding site (RBS) of HA1, with residue 216 [226] (mature H9 numbering used throughout; H3 numbering within brackets) frequently shown to play a critical role (*32*–*34*).

Increasing evidence suggests that the combination and nature of amino acids at additional positions (e.g., 180 [190] and 217 [227]) needs to be determined for assessing receptor binding and zoonotic potential of H9 subtype viruses (*17*). H9N2 viruses commonly have alanine at HA1 position 180 (A180), which correlates with a preference for binding of 3SLN, viruses with glutamic acid (E180) generally show greater binding avidity for 6SLN; and viruses with valine (V180) have been shown to show appreciable binding to 6SLN, together with greater replicative fitness in mammals than viruses with A180 (*17*,*35*,*36*).

The HAs of H9N2 viruses in Pakistan typically contain the RBS residues A180, L216, and I217. A/chicken/Pakistan/UDL-01/2008 (UDL-01/08) and A/chicken/Pakistan/UDL-02/2008 were shown to preferentially bind a variant of the 3SLN receptor analog, which is sulfated at the 6′ position of the penultimate sugar Neu5Ac α2,3 β1–4(6-HSO3)GlcNAc (3SLN(6Su)). Although these viruses contain the classical humanizing residue L216, they show negligible binding to 6SLN in quantitative receptor-binding assays (*16*,*17*).

## Methods

For this study, we conducted influenza surveillance for poultry farms in Pakistan during 2014–2016. Since the late 1990s, H9N2 virus has become enzootic in Pakistan (*37*). In addition, periodic outbreaks of infection with highly pathogenic avian influenza A(H5N1) and A(H7N3) viruses during 1995–2006 have occurred, resulting in intersubtype reassortment events and new viruses, some of which have subsequently spread to become predominant virus strains in the region (*9*,*16*,*38*). We aimed to characterize the genetics and antigenicity of circulating H9N2 viruses in Pakistan and link the molecular basis of differences seen in circulating viruses with zoonotic potential. Specifically, we sought to show that emergence of substitutions at HA1 position 180 are driving increases in virus receptor-binding avidity, including for human-like receptor analogs, and concurrently enabling virus to escape antibody-based immunity (Appendix).

## Results

### Virus Isolation and Phylogenetic Analysis

We collected 1,374 oropharyngeal and cloacal swab specimens from poultry during surveillance of farms for avian influenza viruses in Pakistan during 2014–2016. Of these specimens, 78 (5.7%) were confirmed by hemagglutinin inhibition (HI) assay to be positive for H9, and sequencing data for 55 virus isolates were generated. Next-generation sequencing of isolates yielded 43 complete genomes and 12 partial genomes. No other avian influenza virus subtypes were detected in this study.

Similar to previous results for H9N2 viruses from Pakistan, phylogenetic analysis showed that all HA and NA genes from specimens collected during 2014–2016 continued to belong to the G1 lineage, and all HA genes clustered within the Middle East B clade. Viruses isolated during this study show continued evolution, as shown by phylogenies that demonstrated drift of subclades away from previous isolates (Appendix Figures 1–3). BLASTn (https://blast.ncbi.nlm.nih.gov/Blast.cgi) comparisons for each gene segment of the 2014–2016 viruses against National Center for Biotechnology Information (https://www.ncbi.nlm.nih.gov/) and GISAID (https://www.gisaid.org/) databases showed the closest related viruses to be previous isolates from Pakistan, which had >95% nucleotide homology, suggesting continuous in situ evolution.

Comparison of maximum pairwise nucleotide differences between viruses isolated during 2014–2016 showed variable genetic diversity within each gene segment. Segment 8 (nonstructural) showed the greatest diversity (7% nucleotide difference), and segment 5 (nucleoprotein) showed the least diversity (2% nucleotide difference). In many instances, this diversity could be attributed to a small subset of sequenced viruses: when these segments were removed from the pairwise comparison, virus diversity was reduced. Diversity of gene segments of these outlier viruses that was affected (Appendix Figures 1–3) for gene segments affected from 7% to 2.6% for nonstructural, from 6.1% to 1.1% for polymerase basic 2, and from 6.4% to 2% for HA. Diversity of gene segments remained constant even after removal of those outlier viruses from the pairwise comparison: polymerase basic 1, 4.5%; polymerase acidic, 4.5%; nucleoprotein, 2%; NA, 4.8%; and matrix, 5.5%.

It has previously been shown that H9N2 viruses in Pakistan are reassortants between G1 lineage H9N2 and either H7N3 or clade 2.2 H5N1 subtype viruses (*16*). All internal genes of viruses isolated in this study clustered with the H7N3 virus isolate A/chicken/Pakistan/NARC-100/2004, indicating that this genotype had become predominant in Pakistan. We found no evidence of further intersubtype reassortment.

Using viruses for which we had full-genome sequences and a >2% nt difference cutoff for each segment, we found that viruses isolated during 2014–2016 could be divided into 7 distinct genotypes (Appendix Figures 1–3). Genotypes PK1 and PK2 showed variable intrasubtype reassortment between H9N2 viruses circulating in Pakistan, as shown by phylogenetic incongruence for some genes. Similar patterns of reassortment have been found in Vietnam and China (*15*,*39*). PK3 showed less intrasubtype reassortment than PK1 and PK2.

### Molecular Characteristics of H9N2 Viruses Isolated during 2014–2016

Influenza virus HA requires activation by host proteases at a conserved cleavage site between the HA1 and HA2 subunits. This site is a key determinant of pathogenicity in avian species and describes the switch between low pathogenicity and highly pathogenic avian influenza viruses. Low pathogenicity avian influenza viruses contain monobasic cleavage sites, and highly pathogenic avian influenza viruses contain polybasic cleavage sites (*40*). All H9 HA cleavage sites we sequenced were either KSNR/GLF (10/55) or KSSR/GLF (45/55); viruses containing KSNR/GLF grouped in the PK3 genotype. Previously, HA of H9N2 viruses from Pakistan could be separated into 2 groups containing either KSSR/GLF or RSSR/GLF cleavage sites (*16*), indicating that the KSSR motif has persisted and undergone some evolution to KSNR. These motifs are dibasic and unlikely to be susceptible to activation by endogenous furin-like proteases, leading to classification of these viruses as low pathogenicity avian influenza viruses. However, such dibasic motifs have been described as being susceptible to an extended group of proteases present in a wider range of tissues, potentially enabling these viruses to show increased tissue tropism and pathogenicity (*41*).

All viruses we sequenced in this study contained HA L216, which has been associated with mammalian tropism of H9N2 viruses (*32*). However, recent evidence suggests that L216 alone is a poor marker for human-like receptor-binding preference in H9N2 viruses without considering surrounding amino acids, notably at positions 180 and 217 (*17*). All viruses sequenced contained I217; 52 viruses contained A180 and 3 contained T180.

### Antigenic Characterization of H9N2 Viruses from Pakistan

**Figure 1:**
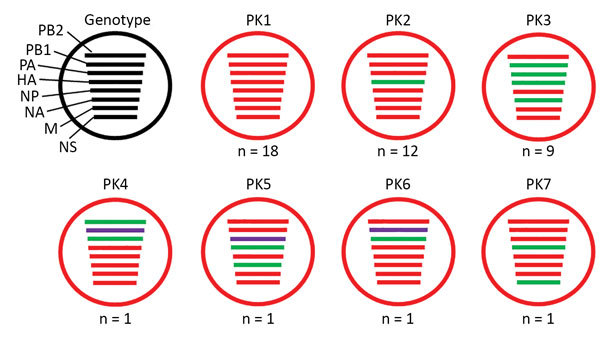
Genotypes of influenza A(H9N2) viruses from Pakistan. Full-genome sequences of 43 contemporary H9N2 avian influenza viruses from Pakistan were used to generate 7 unique genotypes, designated PK1–PK7. Each circle represents a genotype, and n values indicate the total number of viruses assigned to the given genotype. Each line within a circle represents a virus gene segment, and different segment colors between the same gene correspond to a >2% nucleotide difference. Black indicates wild-type virus genes; red, green, and purple indicate mutated genes. HA, hemagglutinin; M, matrix; NA, neuraminidase; NP, nucleoprotein; NS, nonstructural; PA, polymerase acidic; PB, polymerase basic.

To assess antigenic diversity of the 2014–2016 H9N2 virus subtype population (referred to as contemporary viruses), we compared amino acid sequences of HA to identify substitutions in the surface of HA1 (Figure 1). We cloned HA of a 2016 isolate (SKP-827/16) and used it as a backbone to introduce amino acid substitutions by site-directed mutagenesis, which represented the surface diversity of HA1 of the viruses isolated during 2014–2016. Viruses rescued by reverse genetics (RG) differed by only 1 or 2 aa substitutions that reflected diversity seen between individual sequenced isolates. We then assessed HI titers of viruses isolated during 2014–2016 by using chicken postinfection polyclonal antiserum raised against the closely related Pakistan 2008 isolate UDL-01/08. Most viruses were inhibited at titers equivalent to that with the homologous UDL-01/08 virus, but we observed 4-fold reductions in HI titers for viruses containing the HA A180T substitution (Table 1). We observed the same effect when the HA A180T substitution was introduced into wild-type UDL-01/08. Wild-type SKP-827/16 naturally has HA T180, and a 4-fold increase in microneutralization (MNT) titer with antiserum raised against UDL-01/08 was seen when the T180A substitution was introduced, again indicating that this residue alone was responsible for most antigenic diversity in the sequenced viruses. Conversely, for the UDL-01/08 virus, the A180T substitution caused a 3-fold reduction in MNT titer compared with the titer that had the homologous virus (Table 2).

**Table 1:** Change in HI titers of influenza A(H9N2) viruses from Pakistan during 2014–2016 viruses compared to UDL-01/08 virus*

HA backbone and substitution	Fold change in HI titer (titer)†
SKP-827/16	
I116L	– (2,048)
P118S	– (2,048)
S134L	– (2,048)
N135D	– (2,048)
N148S	– (2,048)
A156V	1‡ (4,096)
R162Q + D262N	– (2,048)
G163E	– (2,048)
K164N	1‡ (4,096)
180T	4 (128)
A180T + K164N	4 (128)
A180V	4 (128)
N198D	(2,048)
D262N	(2,048)
S265I	1‡ (4,096)
UDL-01/08	
A180T	4 (128)
A180V	4 (128)

*Postinfection polyclonal antiserum raised in a single chicken inoculated with UDL-01/08 was used in all HI assays. Amino acid substitutions were introduced into a contemporary virus HA backbone (SKP-827/16 with A180) or the UDL-01/08 HA backbone and rescued by reverse genetics. HI titers between contemporary viruses and UDL-01/08 virus were compared. HA, hemagglutinin; HI, hemagglutination inhibition; –, no change.  †The homologous HI titer for UDL-01/08 antiserum was 2,048. ‡Indicates fold decrease compared with UDL-01/08.

**Table 2:** Changes in MNT titers influenza A(H9N2) viruses with HA A/T/V180 substitutions*

HA backbone and substitution	Fold change in MNT titer (titer)†
SKP-827/16	
T180A	4‡ (1,024)
T180V	3§ (4)
UDL-01/08	
A180T	3§ (32)
A180V	>6§ (2)


*MNT assays were conducted by using antiserum raised against UDL-01/08 as before and RG viruses at 100 50% tissue culture infectious doses. Wild-type virus UDL-01/08 was compared to UDL-01/08 HA backbone variants with A180T and A180V substitutions, and wild-type virus SKP-827/16 was compared with SKP-827/16 HA backbone variants with T180A and T180V substitutions. HA, hemagglutinin; MNT, microneutralization. †The homologous MNT titer for UDL-01/08 antiserum was 256. ‡Value indicates fold increase,  §Values indicate fold decrease.

V180 has also been identified as a potential modulator of receptor binding (*35*) because the HA A180T substitution caused a major reduction in HI titer with antiserum raised against UDL-01/08. Thus, we assessed whether the A180V substitution had the same effect. Introduction of the HA V180 substitution into UDL-01/08 and SKP-827/16 RG viruses caused 4-fold greater reductions in HI titer by antiserum raised against UDL-01/08 than for parental RG viruses (Table 1). In a similar fashion, we found a 3-fold greater reduction in MNT titer for virus SKP-827/16 containing the T180V substitution and a >6-fold greater reduction in MNT titer for virus UDL-01/08 containing the A180V substitution than for titers with respective parental RG viruses (Table 2). Our results showed that A180V and A180T substitutions were sufficient to produce virus neutralization escape variants, as assessed by HI and MNT assays, when using postinfection polyclonal antiserum raised against UDL-01/08, which contains HA A180 and which recognizes epitopes in 2 discrete antigenic sites (*42*).

### Determination of Receptor-Binding Avidity by Residue 180

**Figure 2:**
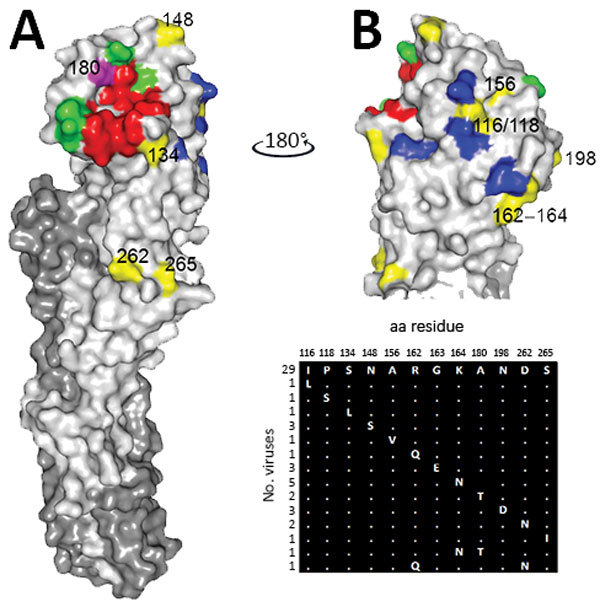
A) H9 HA monomer showing position of each amino acid substitution on the surface of HA1 of contemporary avian influenza A(H9N2) viruses isolated from Pakistan. HA1 is shown in light gray, HA2 in dark gray, receptor binding site in red, previously identified antigenic sites in green and blue (*42**,**43*), and substituted residues identified in this study in yellow. Residue 180 is shown in magenta. B) aa alignment of the HA coding region was used to identify substituted residues within the 2014–2016 population of Pakistan viruses. Shown is the crystal structure of swine H9 hemagglutinin PDB ID:1JSD (*44*), which was drawn by using PyMol software (https://pymol.org/2/). Matrix diagram shows diversity of HA1 surface substitutions and the total number of viruses with a given substitution. Mature H9 numbering is used throughout. aa, amino acid; HA, hemagglutinin.

**Figure 3:**
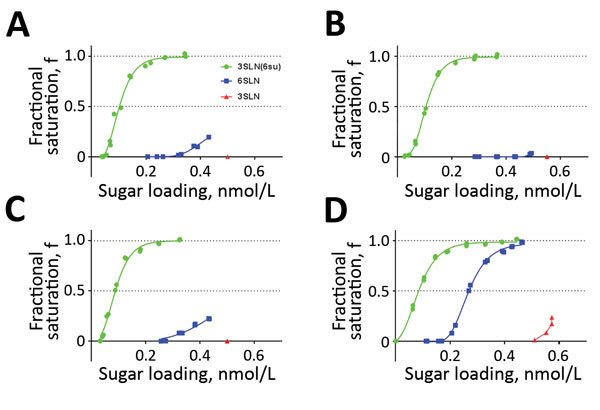
Receptor-binding profiles of wild-type influenza A(H9N2) viruses from Pakistan. Wild-type UDL-01/08 virus SKP and 3 contemporary wild-type viruses were generated by using reverse genetics, and receptor-binding to 3 receptor analogs was assayed by using biolayer interferometry. Sugars tested were 3SLN(6Su) (green), 6SLN (blue), and 3SLN (red). A) H9N2 A/chicken/Pakistan/UDL-01/2008; B) H9N2 A/chicken/LH-55/2014; C) H9N2 A/chicken/SKP-989/2015; D) H9N2 A/chicken/SKP-827/2016.

The HA RBS is located on the head domain of HA1 and is responsible for recognition of sialylated host cell receptors (*27*). To assess the receptor-binding avidity of the 2014–2016 virus population, we identified amino acid substitutions located within the RBS (Figure 2). The only amino acid variation within the RBS of the 2014–2016 viruses was residue A/T180; thus, we generated 3 RG viruses with the HA genes of wild-type SKP-827/16, which naturally contains T180, and viruses A/chicken/LH-55/2014 (LH-55/14) and A/chicken/SKP-989/2015 (SKP-989/15), which contain A180. All 3 viruses have L216 and I217. We used biolayer interferometry (*31*) to characterize the receptor-binding profiles of these viruses and compared them with those of UDL-01/08. LH-55/14 and SKP-989/15, with A180, had UDL-01/08-like receptor-binding profiles, and SKP-827/16, with T180, showed increased receptor-binding avidity for all tested receptor analogs, including an increase in human-receptor binding (Figure 3).

**Figure 4:**
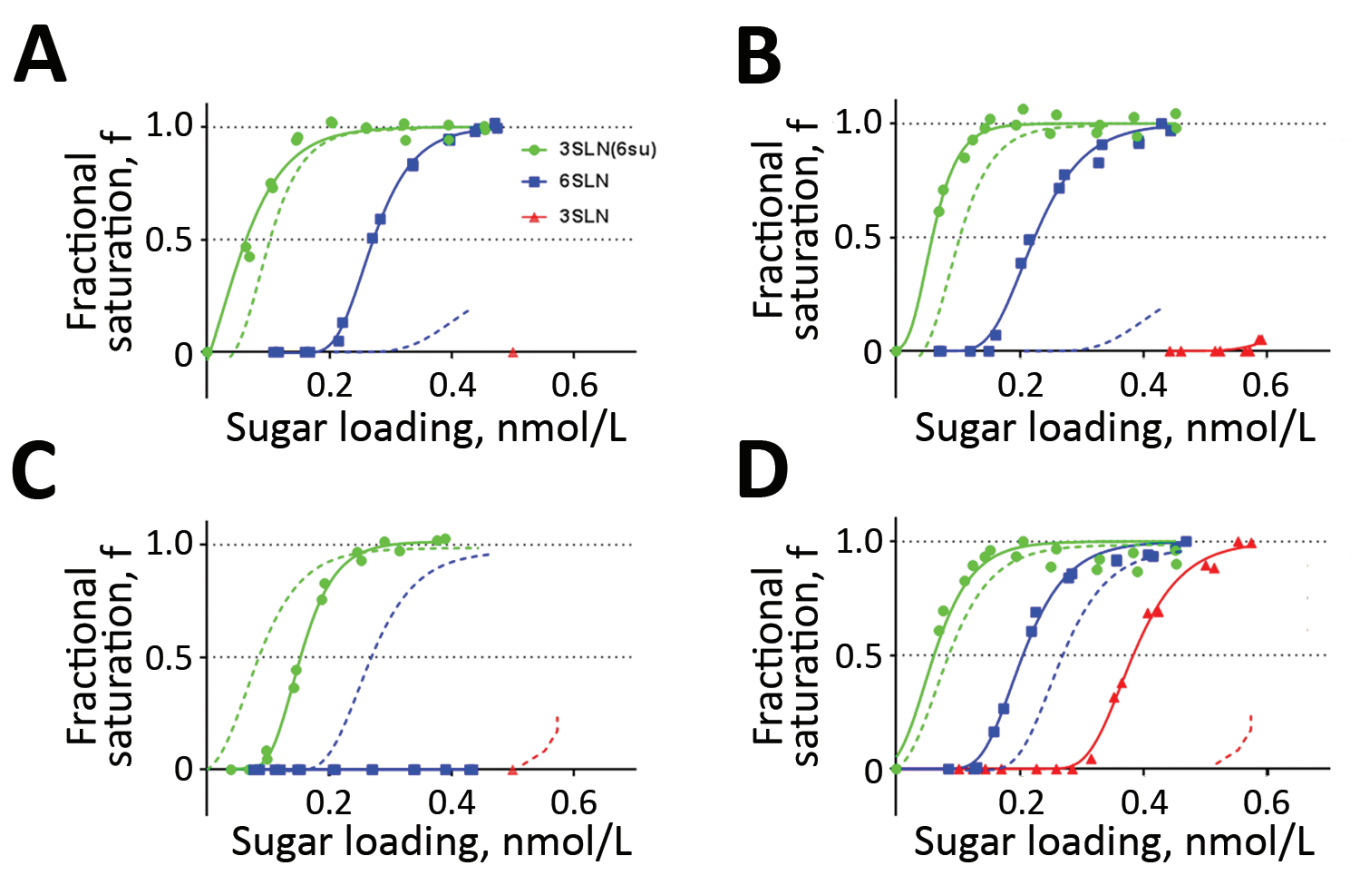
Receptor-binding profiles of influenza A(H9N2) virus isolates from Pakistan with HA residue 180 substitutions. A, B) UDL-01/08 viruses containing A180T/V substitutions: A) H9N2 A/chicken/Pakistan/UDL-10/2008 A180T; B) H9N2 A/chicken/Pakistan/UDL-10/2008 A180V. C, D) SKP-827/16 viruses containing T180A/V substitutions: C) H9N2 A/chicken/Pakistan/SKP-227/2016 T180A; D) H9N2 A/chicken/Pakistan/SKP-227/2016 T180V. Dashed lines indicate binding profiles of wild-type viruses UDL-01/08 with A180 and SKP-827/16 with T180, and solid lines indicate binding profiles of variant viruses. HA, hemagglutinin.

We then used biolayer interferometry on our previously generated UDL-01/08 and SKP-827/16 RG viruses containing A/T/V180 substitutions to define the effect of residue 180. Within the UDL-01/08 backbone, which naturally has A180, A180T caused an ≈50-fold increased binding avidity for 3SLN(6Su) and a >10-fold increased binding to 6SLN. A180V caused an ≈1,000-fold increased binding of 3SLN(6Su) and a >20-fold increased binding of 6SLN (Figure 4, panels A, B). Within the SKP-827/16 backbone, which naturally has T180, T180A caused an ≈30-fold decrease in binding to 3SLN(6Su) and a complete loss of quantifiable binding to 6SLN and 3SLN; T180V caused an ≈130-fold increase in binding to 3SLN(6Su), an ≈4-fold increase in binding to 6SLN, and detectable binding to 3SLN (Figure 4, panels C, D). These results indicated that A180T/V increases the receptor-binding avidity toward all sialylated receptor analogs tested and suggest that the receptor-binding phenotype of wild-type UDL-01/08 could become SKP-827/16-like through substituting A180, and vice versa.

### Requirement of Enhanced Sialidase Activity by Viruses with Enhanced Binding Avidity for Elution from Erythrocytes

**Figure 5:**
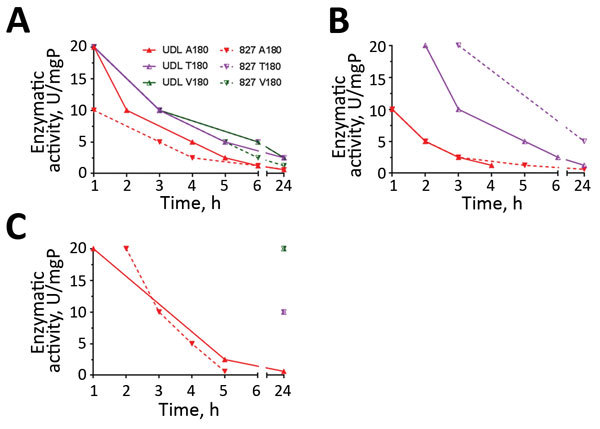
Elution of influenza A(H9N2) viruses (UDL-01/08 and SKP-827/16 A/T/V180) from Pakistan from erythrocytes. Virus elution was recorded at 1, 2, 3, 4, 5, 6, and 24 hours posttreatment with bacterial receptor-destroying enzyme. Points plotted indicate hour at which full loss of hemagglutination was achieved for each concentration of receptor-destroying enzyme. A) Canine erythrocyte elution with UDL and 827 A/T/V180; B) chicken erythrocyte elution with UDL and 827 A/T/V180; C) guinea pig erythrocyte elution with UDL and 827 A/T/V180. mgP, milligram of protein.

For new virions to be released from the surface of infected cells, the NA glycoprotein must have sufficient sialidase activity to outcompete the receptor-binding ability of its cognate HA; this equilibrium is known as HA–NA balance. We investigated A/T180A/T/V variants for their ability to elute from erythrocytes of different species. We used an adapted erythrocyte elution assay whereby different concentrations of bacterial receptor-destroying enzyme (RDE) were added to virus-hemagglutinated erythrocytes and elution monitored for 24 h at 37°C. This assay showed that viruses containing A180 eluted most rapidly, at earlier time points and lower concentrations of RDE than T/V180 viruses, indicating lower receptor avidity (Figure 5). Elution of V180 variants from chicken erythrocytes did not occur even at the highest tested RDE concentration after 24 h of incubation (Figure 5, panel B). Elution from guinea pig erythrocytes was minimal for T180 and V180 variants for which detectable elution only occurred after 24 h at the highest RDE concentrations (Figure 5, panel C). After 24 h incubation, elution from canine erythrocytes was complete for 5 of the test viruses for at least the top 4 RDE concentrations, but the SKP-827/16 T180 variant did not elute even at the highest concentration of RDE (Figure 5, panel A). In agreement with results of receptor-binding assays, we found that viruses with T/V180 have higher avidity toward sialylated receptors that naturally occur on erythrocytes and therefore require more time or better matched sialidase activity to elute efficiently. A control containing virus-hemagglutinated erythrocytes with no RDE treatment was included to show that, by 24 h, viruses containing A180 eluted from guinea pig and chicken erythrocytes but not from canine erythrocytes.

### Effects of Receptor-Binding Avidity on Replication In Vitro

**Figure 6:**
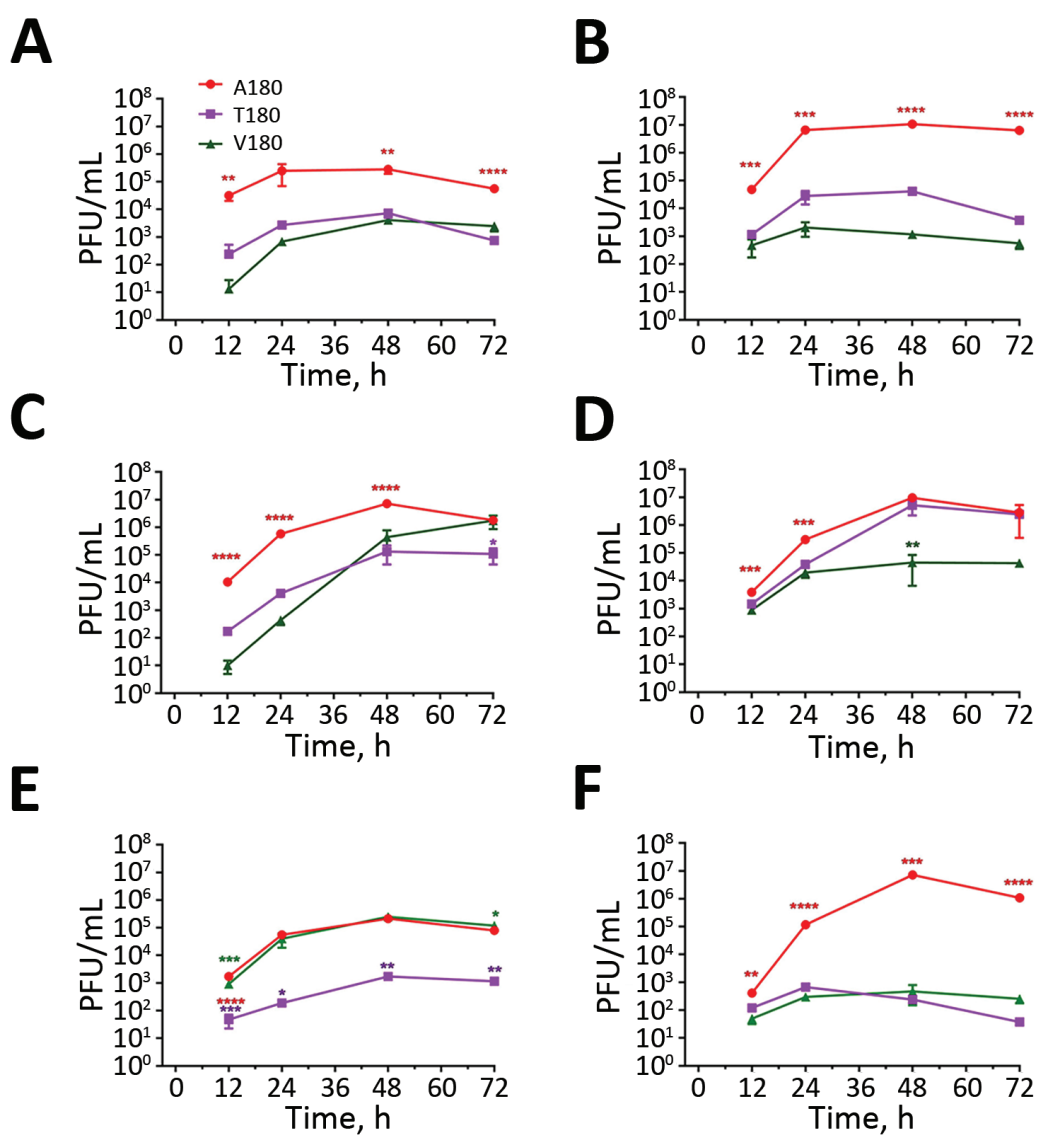
Replication kinetics of influenza A(H9N2) viruses from Pakistan in CKC, MDCK, and MDCK-SIAT1 cells. A, C, E) Replication in CKC, MDCK, and MDCK-SIAT1 cells of UDL-01/08 viruses containing A/T/V180 substitutions; B, D, F) replication in CKC, MDCK, and MDCK-SIAT1 cells of SKP-827/16 viruses containing A/T/V180 substitutions. Virus supernatants were titrated by plaque assay in MDCK cells by using culture supernatants harvested at 12, 24, 48, and 72 hours postinoculation. One-way analysis of variance with multiple comparisons was used to compare virus titers from each time point. A) CKC growth curve: A/chicken/UDL-01/2008 A/T/V/180; B) CKC growth curve: A/chicken/Pakistan/SKP-827/2016 A/T/V/180; C) MDCK growth curve: A/chicken/UDL-01/2008 A/T/V/180; D) MDCK growth curve: A/chicken/Pakistan/SKP-827/2016 A/T/V/180; E) SIAT1 growth curve: A/chicken/Pakistan/UDL-01/2008 A/T/V/180; F) SIAT1 growth curve: A/chicken/Pakistan/ SKP-827/2016 A/T/V/180. *p<0.05; **p<0.01; ***p<0.001; ****p<0.0001).

**Figure 7:**
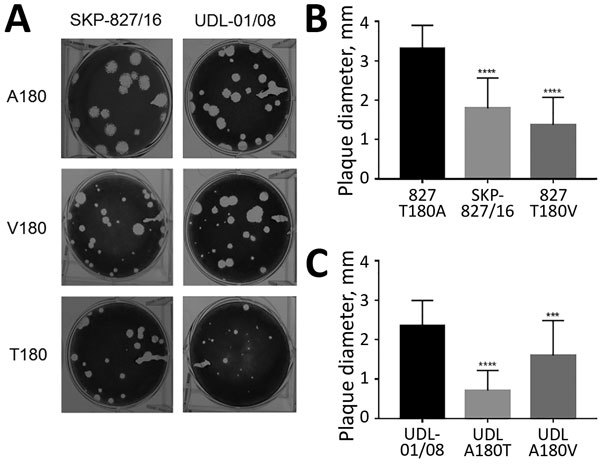
Plaque phenotype of influenza A(H9N2) viruses in UDL-01/08 and SKP-827/16 A/T/V180 variants in MDCK cells. A) Plaque morphology of wild-type UDL-01/08 (containing A180) and SKP-827/16 (containing T180) viruses and variants containing A/T/V180 substitutions. B, C) 30 plaques were selected for each virus, and ImageJ software (https://imagej.nih.gov/ij) was used to measure plaque diameter. Comparisons were conducted between viruses of the same hemagglutinin backbone with different substitutions (A/T/V180). Error bars indicate SEM. ***p<0.001; ****p<0.0001.

To evaluate further the effect on virus replication of the A/T/V180 viruses and variants, we assessed the propagation for each of our viruses by using mammalian and avian cell cultures: MDCKs, MDCK-SIAT1, and primary chicken kidney cells. These cell types express a mixture of α2,3- and α2,6-linked SA, making them suitable for assessing the correlation between growth kinetics and receptor binding (*45*). Viruses with HA T180 or V180 that showed greater receptor-binding avidities replicated to lower titers than did viruses with HA A180 for all cell types (Figure 6). However, at later time points in MDCK-SIAT1 cells, UDL-01/08 A180V replicated to comparable titers with UDL-01/08 A180. These differences in replication efficiency were reflected in MDCK plaque morphology: HA T180 or V180 produced smaller plaque sizes than viruses with HA A180 (Figure 7). This morphology could be seen for the UDL-01/08 and the SKP-827/16 HA backbones in a manner consistent with other avidity increasing amino acid substitutions in H9 virus subtype HAs (*46*).

## Discussion

To effectively control enzootic avian influenza virus infections, it is vital to perform poultry surveillance and virus characterization to inform vaccine composition and use and warn of potential pathogenic or zoonotic threats. The data generated in our study showed that only H9N2 avian influenza viruses were detected among poultry in Pakistan and, although genetically diverse, these viruses were distinct from H9N2 viruses in neighboring countries, implying in situ evolution rather than continuous cross-border spread. Furthermore, H9N2 avian influenza viruses from Pakistan retain internal gene cassettes that are partially derived from an H7N3 virus from Pakistan. Of particular concern is the detection of genotype PK3, which includes viruses that naturally have T180 in HA1, a dibasic motif at the HA cleavage site and truncated polymerase basic 1–F2 (Appendix). We have shown that the A180T substitution causes these viruses to be antigenically distinct from other contemporary H9N2 viruses in Pakistan, possibly leading to escape from vaccine-induced immunity. Moreover, T180 facilitates virus binding to human-like receptors, thus highlighting enhanced zoonotic potential.

Protective immunity generated by infection or conventional inactivated influenza vaccines is mediated though neutralizing antibodies against the major influenza antigen HA. However, viruses are able to evade neutralization by these antibodies through a process known as antigenic drift in human influenza viruses, whereby certain amino acid substitutions in HA are selected that directly prevent antibody binding (*27*,*42*,*47*). Although the mechanism of antigenic drift in avian influenza virus is poorly understood, there is evidence of comparable drivers of antigenic drift, whereby amino acid substitutions around the HA RBS decrease HI antibody titers (*48*). An alternative antibody escape mechanism proposed to be used by influenza viruses is to increase receptor-binding avidity, enabling HA–SA interactions to outcompete inhibition by neutralizing antibodies (*49**,**50*). In this study, we describe avian influenza A(H9N2) viruses that exhibit characteristics of adsorptive mutants in the field, and recently circulating viruses isolated from the field with HA amino acid substitutions that enhance binding avidity toward several different receptor analogs and have reduced HI and MNT titers compared with older viruses, thereby linking receptor-binding avidity to antigenicity. We also showed that HA A180T/V substitutions provide H9N2 viruses with enhanced binding to a human-like 6SLN receptor analog, although these viruses preferentially bind the avian-like 3SLN(6Su) receptor analog. This enhanced binding to 6SLN could enhance zoonotic potential of avian influenza viruses and further highlights the role of HA residue 180 as a marker for mammalian adaptation.

The ability to escape neutralization through enhanced receptor-binding avidity might come at a fitness cost. Virus replication of high-avidity viruses and mutants was attenuated in tested cell lines. An explanation for this finding might be an imbalance in the receptor-binding/cleaving synergy between virus HA and NA; additional HA or NA mutations might be required to rescue the attenuation observed in HA T180 and V180 viruses. Previous studies have investigated the role of residue V180 in mammalian adaptation of H9N2 avian influenza viruses (*20*,*35*,*36*). Teng et al. showed that viruses containing V180 had enhanced replication in mouse lungs and preferential receptor-binding toward α2,6-linked SA compared with α2,3-linked SA, highlighting their inherent ability to bind human-like receptors, which could be possibly attributed to possession of HA L216 (*35*). However, the 3SLN(6Su) receptor analog was not tested by Teng et al., and it is likely that their viruses would have binding preference for this avian-like receptor (*17*). Each of the viruses examined contained a 3-aa deletion in the stalk region of the NA glycoprotein, similar to that previously shown in H9N2 viruses with enhanced NA activity (*25*). All experiments conducted in our study used full-length wild-type UDL-01/08 NA (no stalk deletion), which is naturally paired with UDL-01/08 HA containing A180 and might not be well matched with viruses containing HA T/V180.

Yang et al. (*36*) showed that passage of an avian influenza A(H9N2) virus in differentiated swine airway epithelial cells led to an HA A180V substitution that facilitated α2,6-linked SA binding compare with that of parental virus, which preferentially bound α2,3-linked SA. However, these viruses did not show enhanced replication in porcine cell culture and did not have stalk deletions within the NA glycoprotein (*36*). Chan et al. showed attenuated growth in human lung organ culture of a human H9N2 virus containing HA V180 than in isolates containing D/E180 (*20*). These viruses also did not have any NA stalk deletions. Thus, we postulate that the ability of an H9N2 avian influenza virus containing HA T/V180 to cause efficient infection is at least partially dependent on a stalk deletion within its cognate NA glycoprotein, thus making HA residue 180 a good molecular marker for mammalian adaptation when considered alongside an appropriate NA. We aligned NA glycoproteins from viruses generated in this study and from aforementioned studies, but found that no additional modifications could be correlated with A/T/V180.

In conclusion, we have assessed the pathogenic and zoonotic risks posed by enzootic influenza A(H9N2) viruses in Pakistan by characterizing field isolates. Because of circulation of viruses with potential to escape vaccine-induced immunity, regular updating of vaccines to match circulating strains and protect poultry is needed in Pakistan. Furthermore, isolation of viruses from the G1 lineage with enhanced human receptor-binding avidity warrants continued surveillance for poultry and persons working with or near poultry.

Appendix Additional information on association of increased receptor-binding avidity of influenza A(H9N2) viruses with escape from antibody-based immunity and enhanced zoonotic potential.
