# Supplementary material for: Association of Increased Receptor-Binding Avidity of Influenza A(H9N2) Viruses with Escape from Antibody-Based Immunity and Enhanced Zoonotic Potential
Source: Emerg Infect Dis. 2019 Jan;25(1):63–72. doi: 10.3201/eid2501.180616 (PMC6302589; doi:10.3201/eid2501.180616)
Supplement: Appendix — Additional information on association of increased receptor-binding avidity of influenza A(H9N2) viruses with escape from antibody-based immunity and enhanced zoonotic potential. [file 18-0616-Techapp-s1.pdf]

Article DOI: https://doi.org/10.3201/eid2501.180616

Association of Increased Receptor-Binding Avidity of Influenza A(H9N2) Viruses with Escape from Antibody-Based Immunity and Enhanced Zoonotic Potential

Appendix

Polymerase basic protein 1(PB1)–F2 is encoded by influenza virus genome segment 2 and is translated from a downstream start codon containing a strong Kozak sequence in the +1 reading frame from PB1 mRNA (*1*). Viruses encode either full-length $\geq$87/90 aa or truncated $\leq$79 aa PB1-F2 proteins. Full-length PB1-F2 has been shown to facilitate prolonged virus shedding and lower pathogenicity in influenza A(H9N2) virus–infected chickens, and truncated PB1-F2, observed in naturally occurring highly pathogenic avian influenza A(H5N1) viruses causes enhanced pathogenicity in mice (*2*,*3*). Of 46 sequenced PB1-F2 genes in this study, 35 were truncated (57 aa) when compared with previous H9N2 subtype avian influenza viruses from Pakistan, most (27/29 viruses) of which were full length. Historically, most early H5N1 subtype viruses had full length PB1-F2, but recently, greater numbers of variants with truncated PB1-F2 have been observed (*3*), a situation that mirrors what is being seen in H9N2 subtype viruses in Pakistan.

Materials and Methods

Sample Collection, Screening, and Virus Isolation

Monthly surveillance for H9N2 subtype avian influenza virus was conducted on poultry farms in 24 districts throughout 2 provinces of Pakistan during December 2014–December 2016 by the University of Veterinary and Animal Sciences (Lahore, Pakistan). Oropharyngeal and cloacal swab specimens were taken from birds and stored in virus transport medium and maintained in a cold chain for transportation to University of Veterinary and Animal Sciences where they were stored at –80°C. Embryonated hen eggs were inoculated with virus transport medium from swabs specimens, and allantoic fluid was recovered and screened for influenza A

virus by hemagglutination assay. Subtyping of hemagglutinin (HA)–positive samples was conducted by hemagglutinin inhibition assay using H5, H7, and H9 reference antisera. In preparation for sequencing, virus RNA was extracted from allantoic fluid by using the QIAamp Viral RNA Mini Kit (QIAGEN, https://www.qiagen.com/us/). Multisegment reverse transcription PCR was conducted as described (*4*) by using the SuperScript III One-Step RT-PCR Kit *(*Life Technologies, https://www.thermofisher.com/us/en/home/brands/life-technologies.html) and the MBTUni12/13 universal primer set. Sequencing data were generated for H9N2 subtype avian influenza viruses from 46 broiler chickens, 3 breeder chickens, 2 local breed Desi chickens, 1 layer chicken, 2 ducks, and 1 partridge.

Next-Generation Sequencing

Next-generation sequencing was conducted by using preamplified reverse transcription PCR products, which were used to generate DNA libraries by using the Nextera XT DNA Library Prep Kit (Illumina, https://www.illumina.com/) with 96 dual-index barcodes. Pooled libraries were sequenced on an Illumina MiSeq, and resultant sequencing reads used to generate virus genomes by Templated assembly in SeqMan NGen and SeqMan Pro (DNASTAR, https://www.dnastar.com/). Nucleotide sequences obtained in this study have been submitted to GenBank under accession numbers PB2 (MH180764–MH180812), PB1 (MH180707–MH180752), polymerase acidic (MH180653–MH180702), HA (MH180385–MH180439), nucleoprotein (MH180546–MH180599), neuraminidase (NA) (MH180493–MH180544), matrix (MH180440–MH180492), and nonstructural protein (MH180600–MH180652).

Phylogenetic and Molecular Analysis

Nucleotide and deduced amino acid sequences were aligned and analyzed by using BioEdit (http://www.mbio.ncsu.edu/BioEdit/bioedit.html), and maximum-likelihood phylogenetic trees were estimated by using RaxML version 8.2X (https://sco.h-its.org/exelixis/software.html), followed by annotation with amino acid substitutions defining nodes and individual virus gene products by using treesub (https://github.com/tamuri/treesub/blob/master/README.md). Trees were visualized by using FigTree (http://tree.bio.ed.ac.uk/software/figtree/) and highlighted by using Adobe Illustrator CC 2015.3 (http://www.adobe.com/uk/products/illustrator/features.html). Reference virus sequences analyzed alongside sequences generated in this study were downloaded from the NCBI (https://www.ncbi.nlm.nih.gov/) and GISAID (https://www.gisaid.org/) databases. Genotyping

was conducted by using a 2% nucleotide difference cutoff to indicate genetically divergent virus gene segments as described (*5*).

Virus Reverse Genetics and Mutagenesis

Reverse-genetics (RG) viruses were generated from either 8 or 9 cotransfected bidirectional reverse genetics plasmids as described (*2,6*). Viruses were rescued from the named HA plasmid, the NA of UDL-01/08, and either the 6 internal genes of UDL-01/08 or the 6 internal genes of A/Puerto Rico/8/34 (PR8). Antigenic and receptor-binding assays were conducted by using RG viruses with PR8 internal genes, and growth curves were constructed by using RG viruses with UDL-01/08 internal genes. Site-directed mutagenesis was used to introduce encoded amino acid substitutions into RG plasmids containing the HA of either UDL-01/08 or SKP-827/16 by using the QuickChange Lightning Kit (Agilent, https://www.agilent.com/). After each virus had been rescued and cultivated in embryonated hens eggs (VALO, http://www.valobiomedia.com/35.vaccine-eggs.html), full-length HA and NA genes were sequenced by using Sanger sequencing at Source BioScience (https://www.sourcebioscience.com/) to confirm that no additional mutations had been introduced (*7*).

Antigenic Characterization and Microneutralization

Viruses were titrated by hemagglutination assay and then diluted to 4 HA units and used in HI assays as described (*8*). In brief, 4 HA units of each virus was incubated with antiserum, raised in a single specific pathogen-free chicken infected with UDL-01/08 virus (*7*), which had been serially diluted 2-fold in phosphate-buffered saline (PBS) for 1 hour and then incubated with 1% chicken red blood cells (RBCs) diluted in PBS for 1 hour. HI titer is expressed as the reciprocal of the highest serum dilution at which hemagglutination was completely inhibited.

For the microneutralization assays, viruses were first titrated by using 50% tissue culture infectious doses as described by using the Spearman-Kärber formula (*8*). One hundred 50% tissue culture infectious doses of each virus was added in triplicate to a dilution series of heat-inactivated antiserum raised against UDL-01/08, incubated for 1 hour at 37°C, and then inoculated onto confluent MDCK cells for 1 hour at 37°C. Cells were then washed in PBS and overlaid with virus growth medium (serum-free Dulbecco minimal essential medium plus 2

μg/mL of tosylamide-2-phenylethyl chloromethyl ketone trypsin). After 72 hours, cells were washed with PBS, fixed, and stained with crystal violet.

Virus Purification and Biolayer Interferometry

Virus purification and biolayer interferometry were conducted as described (*7*,*9*). In brief, virus-infected allantoic fluid was harvested from embryonated hen eggs and centrifuged at 27,000 rpm for 2 hours. Pelleted virus was resuspended in PBS and centrifuged at 27,000 rpm for 2 hours through a continuous 30%–60% sucrose gradient, then isolated from a fraction within the sucrose gradient and centrifuged again at 27,000 rpm for 2 hours. Purified virus was used in biolayer interferometry assays on an Octet RED Bio-Layer Interferometer (Pall ForteBio, https://www.fortebio.com/). We measured virus binding to sialic acid analogs α2,6-sialyllactosamine (6SLN), α2,3-sialyllactosamine (3SLN), Neu5Ac α2,3 gal β1–4(6-HSO_3) and GlcNAc (3SLN(6Su)) (GlycoNZ, Auckland, New Zealand). Relative estimated dissociation constants (K_D) were calculated by using GraphPad Prism version 6 (GraphPad, https://www.graphpad.com/) as described (*9*).

Growth Curves

For in vitro growth curves in MDCK cells and CKC cells, virus was inoculated in triplicate at a multiplicity of infection of 0.01 for 1 hour before washing with PBS and overlaying with virus growth medium for MDCK cells and EMEM supplemented with 0.7% (wt/vol) bovine serum albumin and 10% tryptose phosphate buffer for CKC cells. Culture supernatants were taken at 12, 24, 48 and 72 hours postinfection and titrated by plaque assay in MDCK cells (*8*). All viruses used in growth curves contained the NA and internal genes from UDL-01/08 and the HA of either wild-type or variant UDL-01/08 and SKP-827/16 viruses.

Sialidase Assay

A virus elution assay was used to determine the different NA concentrations required for virus elution from chicken, canine, and guinea pig RBCs over 24 hours. Virus was diluted in PBS to 4 HA units and incubated with 1% RBCs for 1 hour in v-bottom 96-well plates to enable hemagglutination to occur. A 2-fold dilution series of the receptor-destroying enzyme (RDE), bacterial NA from *Vibrio cholerae* (Sigma-Aldrich, https://www.sigmaaldrich.com/united-kingdom.html), was prepared at 20, 10, 5, 2.5, 1.25, and 0.62 units/mg protein (lot no. 126M4092v) and added to each well containing hemagglutinated virus. Plates were incubated for

24 hours at 37°C, and total virus elution was recorded every hour for the first 6 hours and again at 24 hours. The time at which total elution of virus from RBCs was achieved at each RDE concentration was recorded and plotted. Controls included no-virus wells and no-RDE wells.

References

1. Chen W, Calvo PA, Malide D, Gibbs J, Schubert U, Bacik I, et al. A novel influenza A virus mitochondrial protein that induces cell death. Nat Med. 2001;7:1306–12. PubMed http://dx.doi.org/10.1038/nm1201-1306

2. James J, Howard W, Iqbal M, Nair VK, Barclay WS, Shelton H. Influenza A virus PB1-F2 protein prolongs viral shedding in chickens lengthening the transmission window. J Gen Virol. 2016;97:2516–27. PubMed http://dx.doi.org/10.1099/jgv.0.000584

3. Kamal RP, Kumar A, Davis CT, Tzeng W-P, Nguyen T, Donis RO, et al. Emergence of highly pathogenic avian influenza A(H5N1) virus PB1–F2 variants and their virulence in BALB/c mice. J Virol. 2015;89:5835–46. PubMed http://dx.doi.org/10.1128/JVI.03137-14

4. Zhou B, Donnelly ME, Scholes DT, St George K, Hatta M, Kawaoka Y, et al. Single-reaction genomic amplification accelerates sequencing and vaccine production for classical and Swine origin human influenza a viruses. J Virol. 2009;83:10309–13. PubMed http://dx.doi.org/10.1128/JVI.01109-09

5. Thuy DM, Peacock TP, Bich VT, Fabrizio T, Hoang DN, Tho ND, et al. Prevalence and diversity of H9N2 avian influenza in chickens of Northern Vietnam, 2014. Infect Genet Evol. 2016;44:530–40. PubMed http://dx.doi.org/10.1016/j.meegid.2016.06.038

6. Hoffmann E, Neumann G, Kawaoka Y, Hobom G, Webster RG. A DNA transfection system for generation of influenza A virus from eight plasmids. Proc Natl Acad Sci U S A. 2000;97:6108–13. PubMed http://dx.doi.org/10.1073/pnas.100133697

7. Peacock TP, Benton DJ, James J, Sadeyen J-R, Chang P, Sealy JE, et al. Immune escape variants of H9N2 influenza viruses containing deletions at the haemagglutinin receptor binding site retain fitness in vivo and display enhanced zoonotic characteristics. J Virol. 2017;91:e00218–17. PubMed http://dx.doi.org/10.1128/JVI.00218-17

8. World Health Organization. Manual for the laboratory diagnosis and virological surveillance of influenza. WHO Global Influenza Surveillance Network. Geneva: The Organization; 2011.

9. Peacock TP, Benton DJ, Sadeyen J-R, Chang P, Sealy JE, Bryant JE, et al. Variability in H9N2 haemagglutinin receptor-binding preference and the pH of fusion. Emerg Microbes Infect. 2017;6:e11. PubMed http://dx.doi.org/10.1038/emi.2016.139

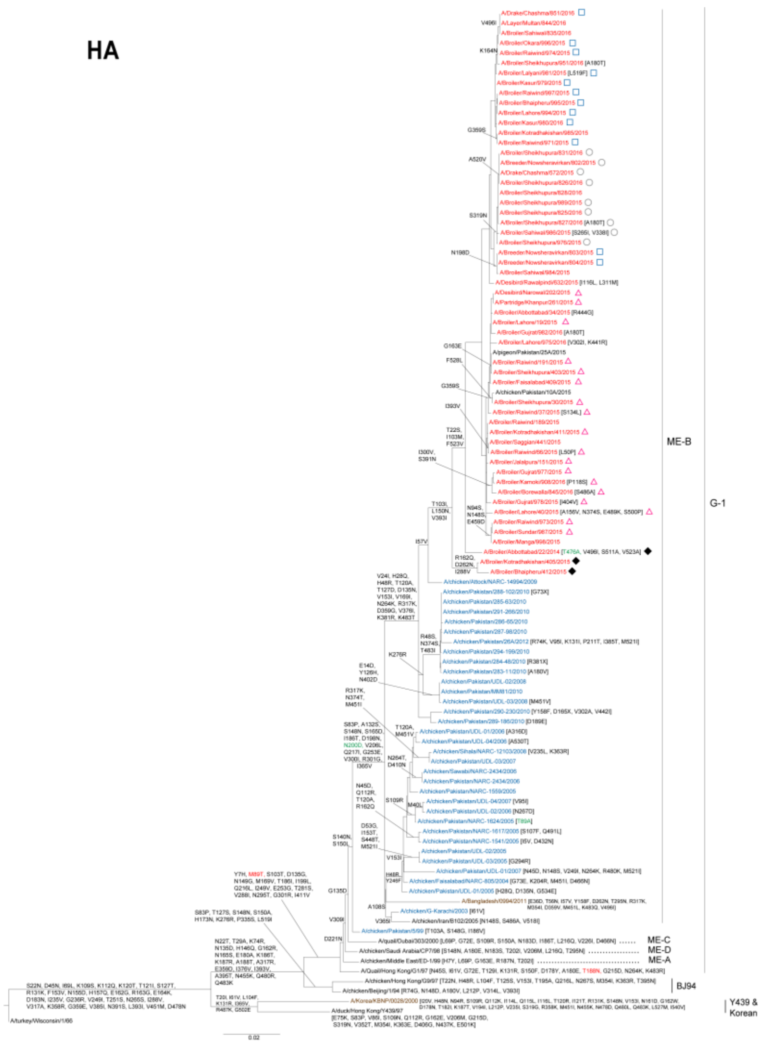

Appendix Figure 1. Phylogenetic tree of HA genes of influenza A(H9N2) viruses from Pakistan. Shown are lineage and clade representative viruses and other sequences downloaded from NCBI (https://www.ncbi.nlm.nih.gov/) and GISAID (https://www.gisaid.org/) databases (black: Y439 and Korean; Beijing/1/94 (BJ94); Middle Eastern A (ME-A), B (ME-B), C (ME-C) and D (ME-D); all viruses but those in the BJ94 and Y349 and Korean lineages fall in the generic G1 clade), H9N2 viruses isolated from humans (brown), H9N2 viruses isolated during 1999–2012 from Pakistan (blue), and 2014–2016 H9N2 viruses isolated in this study (red). Empty markers to the right of branches indicate the 3 most abundant contemporary Pakistan genotypes (for viruses with complete genome sequences): PK1 (pink triangles); PK2 (blue squares); and PK3 (gray circles). Black diamonds indicate viruses with <98% nucleotide homology compared with other 2014–2016 viruses from Pakistan. Amino acid substitutions resulting in loss (green text) or gain (red text) of potential N-linked glycosylation sites are indicated, and polymorphic substitutions are indicated with X (e.g., HA G73X). Scale bar indicates nucleotide substitutions per site. HA, hemagglutinin.

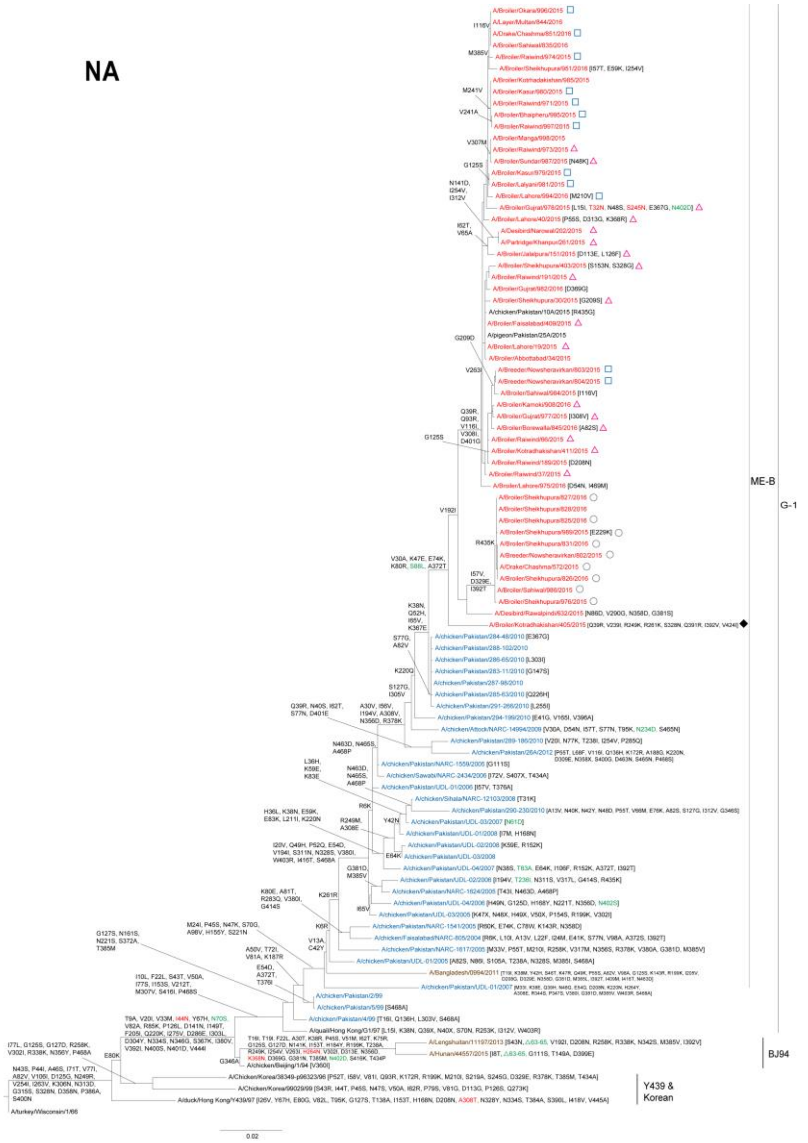

Appendix Figure 2. Phylogenetic tree of NA genes of Influenza A(H9N2) viruses from Pakistan. Shown are lineage and clade representative viruses and other sequences downloaded from NCBI (https://www.ncbi.nlm.nih.gov/) and GISAID (https://www.gisaid.org/) databases (black: Y439 and Korean; Beijing/1/94 (BJ94); Middle Eastern A (ME-A), B (ME-B), C (ME-C) and D (ME-D); all viruses but those in the BJ94 and Y349 and Korean lineages fall in the generic G1 clade), H9N2 viruses isolated from humans (brown), H9N2 viruses isolated between 1999 and 2012 from Pakistan (blue), and 2014–2016 H9N2 viruses isolated in this study (red). Empty markers to the right of branches indicate the 3 most abundant contemporary Pakistan genotypes (for viruses with complete genome sequences): PK1 (pink triangles); PK2 (blue squares); and PK3 (gray circles). Black diamonds indicate viruses with <98% nucleotide homology compared with other 2014–2016 viruses from Pakistan. Amino acid substitutions resulting in loss (green text) or gain (red text) of potential N-linked glycosylation sites are indicated while polymorphic substitutions are indicated with X (e.g., HA G73X). In the NA phylogeny, Δ63–65 indicates

deletion of 3 amino acids in the NA glycoprotein stalk region. Scale bar indicates nucleotide substitutions per site. NA, neuraminidase

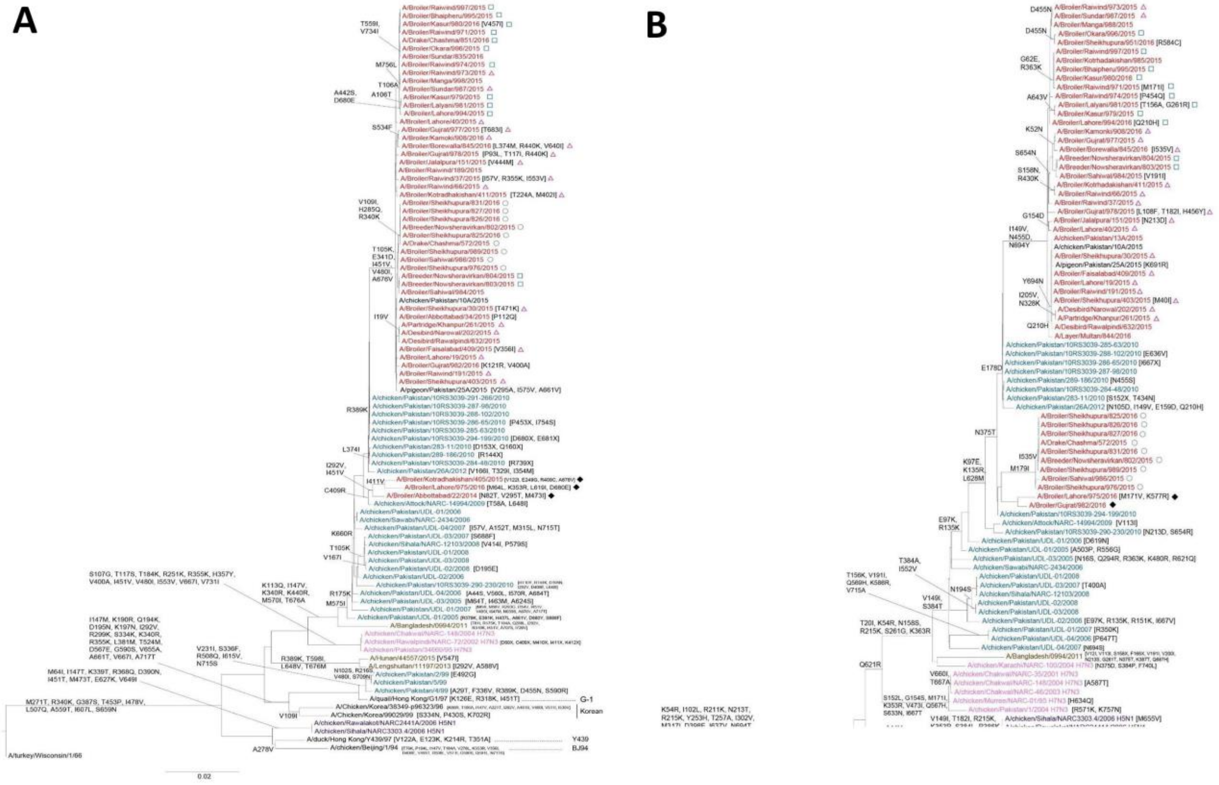

C

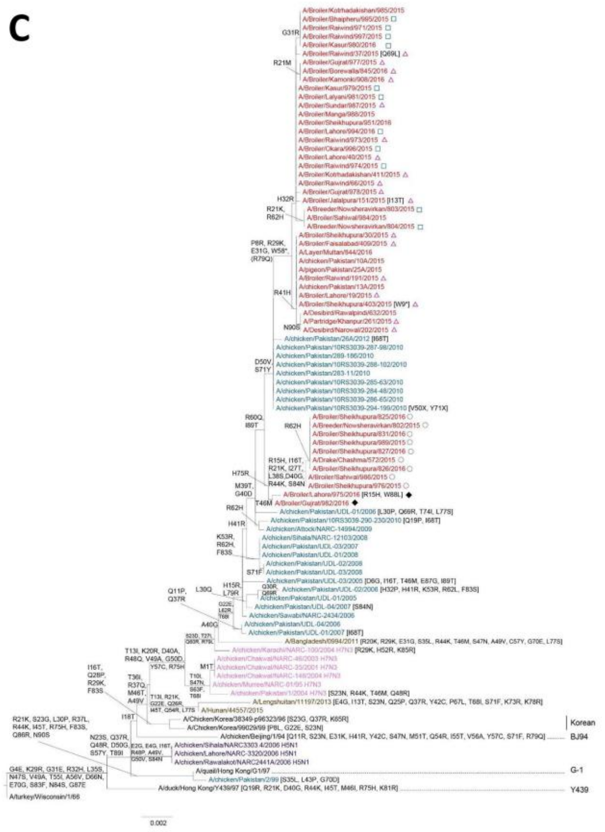

D

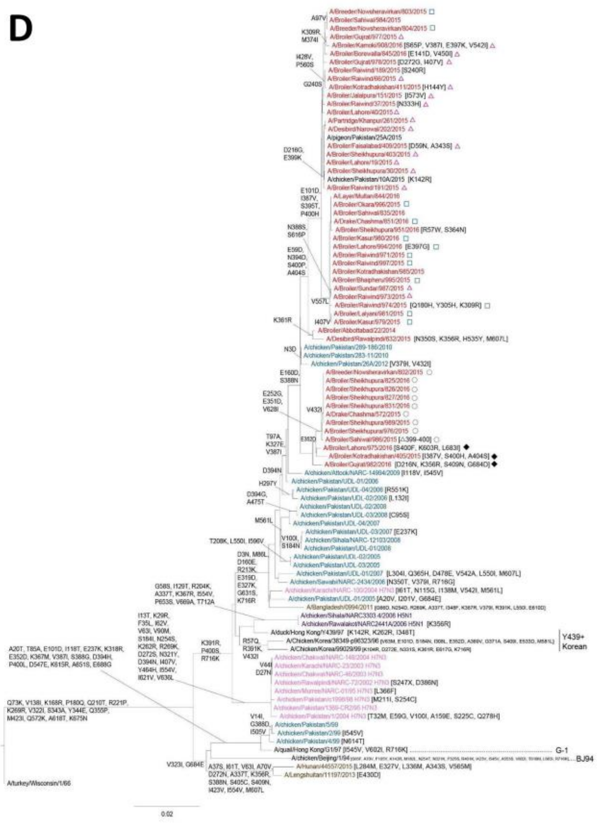

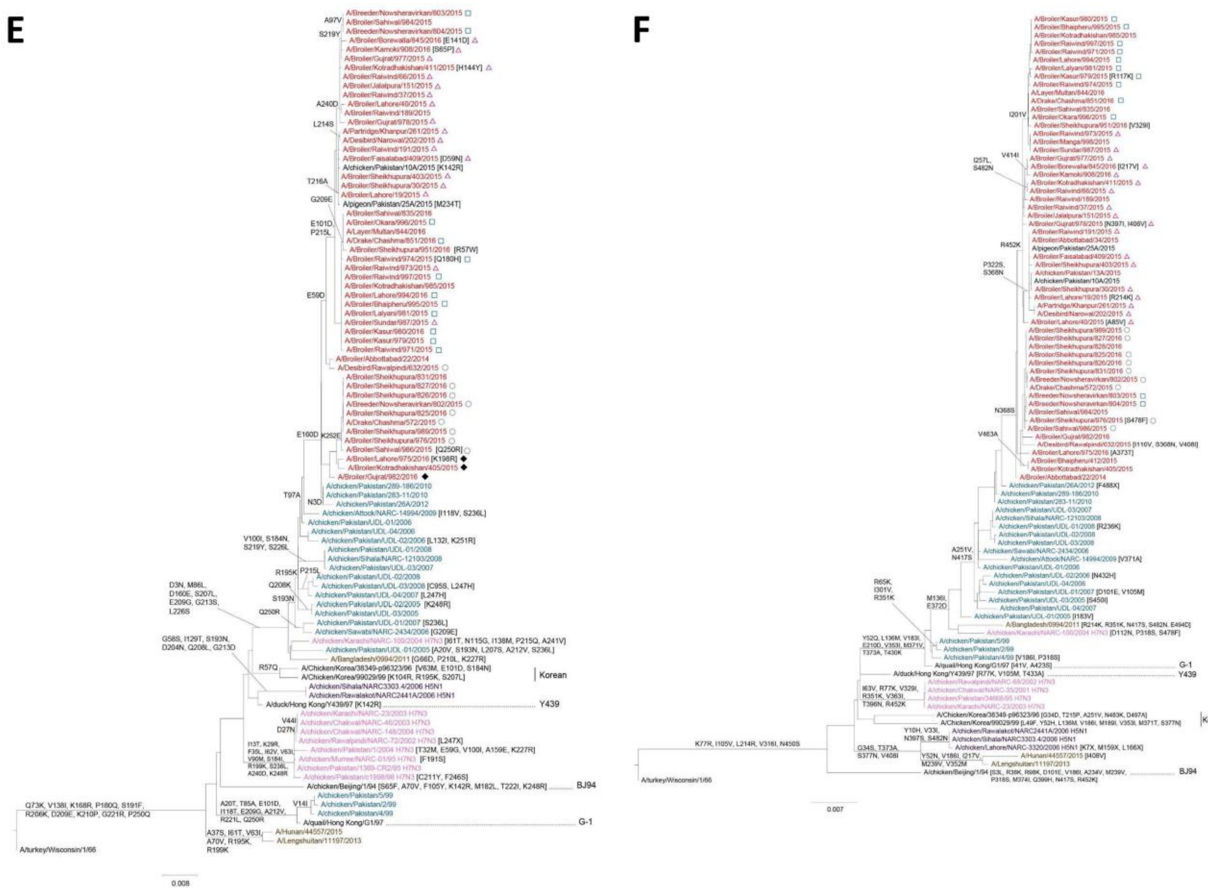
E
F

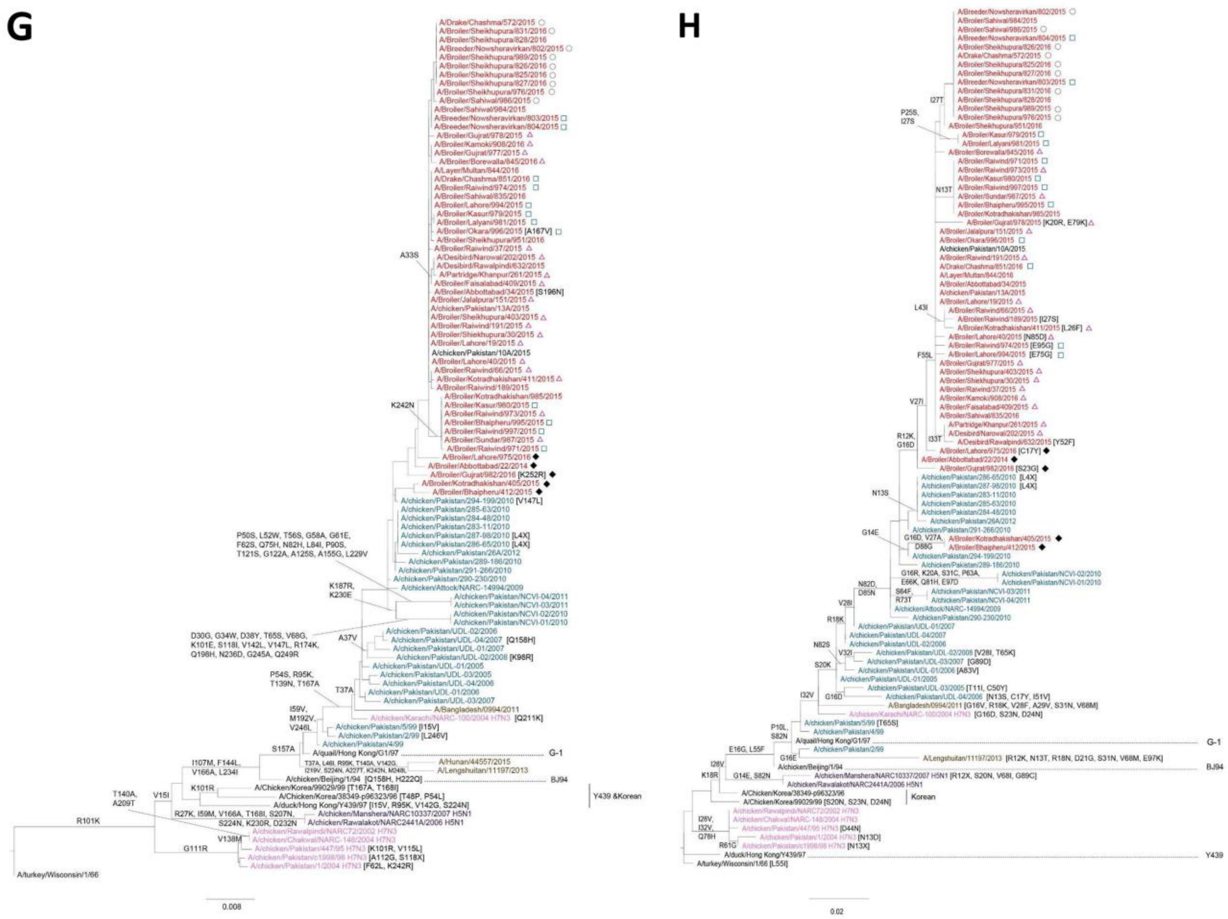
G
0.008
H
0.02

I

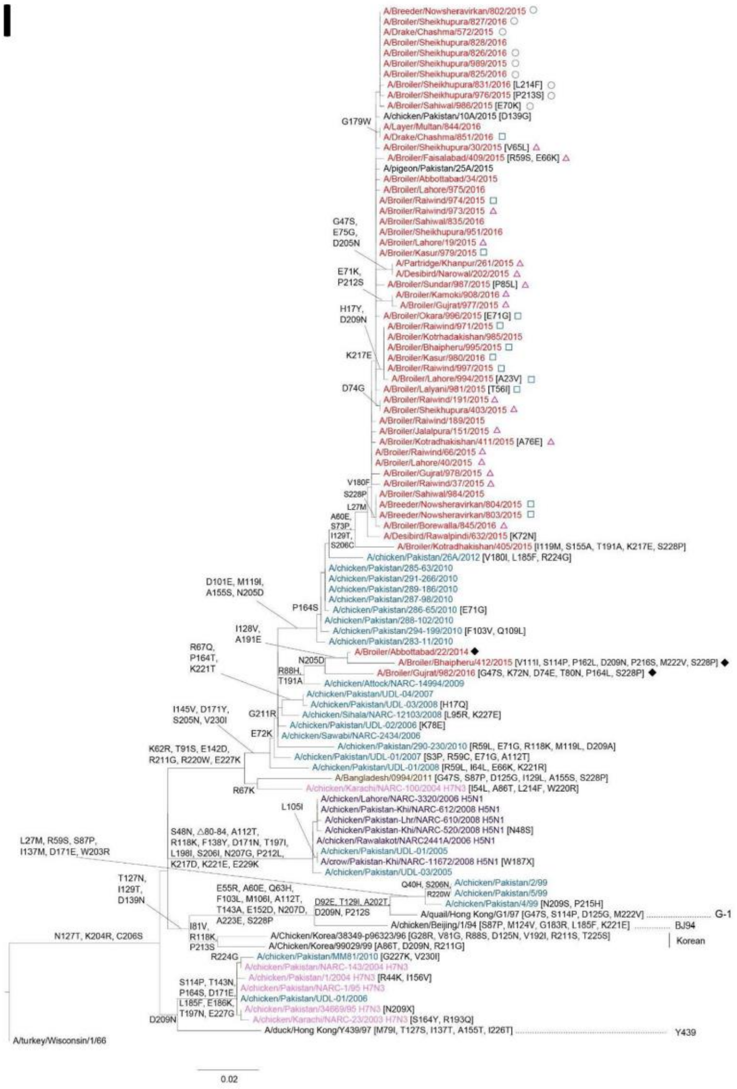

Appendix Figure 3. Phylogenetic analyses of internal genes of avian influenza A(H9N2) virus by using encoded protein open reading frames. A) PB2; B) PB1; C) PB1-F2; D) PA; E) PAX; F) NP; G) M1; H) M2; I) NS1; J) NEP. Shown are lineage and clade representative viruses and other sequences downloaded from NCBI (https://www.ncbi.nlm.nih.gov/) and GISAID (https://www.gisaid.org/) databases: (black); Y439 and Korean; A/Beijing/1/94 (BJ94); A/quail/Hong Kong G1/97 (G-1); all viruses but A/turkey/Wisconsin/1/66 and those in BJ94 and Y349 and Korean lineages fall in the generic G1 clade); H9N2 viruses isolated from humans (brown); H9N2 viruses isolated during 1999–2012 from Pakistan (blue); and 2014–2016 H9N2 viruses isolated in this study (red). Empty markers to the right of branches indicate the 3 most abundant contemporary Pakistan genotypes (for viruses with complete genome sequences): PK1 (pink triangles); PK2 (blue squares); and PK3 (gray circles). For individual genes, black diamonds indicate viruses with <97% nucleotide homology compared with other viruses from Pakistan isolated during 2014–2016. Polymorphic amino acid substitutions are indicated with X (e.g., PB2 P453X). Amino acid deletions (∆) are present in PA (∆ 399–400) of A/Broiler/Sahiwal/986/2015 and NS1 (∆ 80–

84) of a group of viruses from Pakistan isolated during 2005–2008. Many PB1-F2 open reading frames carry premature termination codons (*), notably those with PK1 and PK2 genotypes, which terminate at residue 57 (W58*). Scale bars indicate nucleotide substitutions per site. M, matrix; NEP, nuclear export protein; NP, nucleoprotein; NS, nonstructural; PA, polymerase acidic; PAX, polymerase acidic frameshift protein; PB, polymerase basic.
